# Complete Genome Sequence of the Triclosan- and Multidrug-Resistant *Pseudomonas aeruginosa* Strain B10W Isolated from Municipal Wastewater

**DOI:** 10.1128/genomeA.01489-16

**Published:** 2017-01-19

**Authors:** Chuanqing Zhong, Matthew Nelson, Guangxiang Cao, Michael J. Sadowsky, Tao Yan

**Affiliations:** a School of Municipal and Environmental Engineering, Shandong Jianzhu University, Jinan, China; b Department of Civil and Environmental Engineering, University of Hawaii at Manoa, Honolulu, Hawaii, USA; c Biotechnology Institute and Department of Soil, Water, and Climate, University of Minnesota, St. Paul, Minnesota, USA; d Shandong Medicinal Biotechnology Centre, Shandong Academy of Medical Sciences, Jinan, China

## Abstract

Here, we report the complete genome sequence of the triclosan- and multidrug-resistant *Pseudomonas aeruginosa* strain B10W, obtained from municipal wastewater in Hawaii. The bacterium has a 6.7-Mb genome, contains 6,391 coding sequences and 78 RNAs, with an average G+C content of 66.2 mol%.

## GENOME ANNOUNCEMENT

*Pseudomonas aeruginosa* is an important opportunistic pathogen that exhibits high intrinsic resistance to biocides and antibiotics (1). Triclosan is a biocide widely used in both clinical and consumer product settings (2). Recent identification of a cellular target of triclosan, the enoyl-acyl carrier protein reductase (ENR) (FabI) (3, 4), has cast significant doubt on its usage as a biocide. Previous studies have shown that *P. aeruginosa* is intrinsically resistant to triclosan due to the activities of multiple efflux pumps (5) and the presence of an alternative ENR, FabV, that is highly resistant to triclosan (6).

*P. aeruginosa* is ubiquitously present in the environment, including municipal wastewater (7). Although the biogeography of fluorescent *Pseudomonas* strains from soils has been examined in some detail (8), few studies have examined environmental *P. aeruginosa* isolates (9). The *Pseudomonas aeruginosa* strain B10W was isolated from municipal wastewater collected in Honolulu, Hawaii, USA, using LB agar supplemented with triclosan (10 μg/mL). Antibiotic susceptibility tests showed that this bacterium exhibited resistance to tetracycline (20 µg/mL), chloramphenicol (10 μg/mL), kanamycin (100 μg/mL), ampicillin (100 μg/mL), and nalidixic acid (20 μg/mL). The B10W genomic DNA was extracted from overnight culture using a GenElute bacterial genomic DNA kit (Sigma). The genomic DNA was subjected to PacBio SMRT cell sequencing. The sequence reads were assembled into a single 6.7-Mb contig using HGAP assembly 3. The contig was verified as circular using Gepard version 1.3.1. The circular contig was reassembled to polish the assembly. The mean sequencing coverage was 118.8-fold, with accuracy greater than 99.99%.

The automated annotation of the B10W genome was performed using the RASTtk server (10), which predicted 6,391 coding sequences, including 78 RNAs. Several multidrug efflux pumps of the resistance nodulation cell division (RND) family, which were previously detected in *P. aeruginosa* strains, were detected in the B10W genome. These included MexAB-OprM, MexCD-OprJ, and MexEF-OprN (11). The MexJK (12) and TriABC-OpmH (13) efflux pumps, however, were not detected. Other efflux pumps detected include CmeABC, which was previously found in *Campylobacter jejuni* (14), and the CzcC superfamily for heavy metal resistance, which was frequently detected in soil bacteria (15). Sequence-based searches done using the Resfinder (16) detected five acquired resistance genes in the B10W genome, encoding resistance to chloramphenicol, aminoglycoside, beta-lactams, and fosfomycin.

### Accession number(s).

The complete nucleotide sequence of the *P. aeruginosa* strain B10W genome was deposited in GenBank under accession number CP017969.

## References

[1] Murray JL, Kwon T, Marcotte EM, Whiteley M 2015 Intrinsic antimicrobial resistance determinants in the superbug *Pseudomonas aeruginosa*. mBio 6:e01603-15. doi:10.1128/mBio.01603-15. 26507235 PMC4626858

[2] Jones RD, Jampani HB, Newman JL, Lee AS 2000 Triclosan: a review of effectiveness and safety in health care settings. Am J Infect Control 28:184–196. doi:10.1067/mic.2000.102378. 10760227

[3] McMurry LM, Oethinger M, Levy SB 1998 Triclosan targets lipid synthesis. Nature 394:531–532. doi:10.1038/28970. 9707111

[4] Heath RJ, Yu YT, Shapiro MA, Olson E, Rock CO 1998 Broad spectrum antimicrobial biocides target the FabI component of fatty acid synthesis. J Biol Chem 273:30316–30320. doi:10.1074/jbc.273.46.30316. 9804793

[5] Chuanchuen R, Beinlich K, Hoang TT, Becher A, Karkhoff-Schweizer RR, Schweizer HP 2001 Cross-resistance between triclosan and antibiotics in *Pseudomonas aeruginosa* is mediated by multidrug efflux pumps: exposure of a susceptible mutant strain to triclosan selects *nfxB* mutants overexpressing MexCD-OprJ. Antimicrob Agents Chemother 45:428–432. doi:10.1128/AAC.45.2.428-432.2001. 11158736 PMC90308

[6] Zhu L, Lin J, Ma J, Cronan JE, Wang H 2010 Triclosan resistance of *Pseudomonas aeruginosa* PAO1 is due to FabV, a triclosan-resistant enoyl-acyl carrier protein reductase. Antimicrob Agents Chemother 54:689–698. doi:10.1128/AAC.01152-09. 19933806 PMC2812149

[7] Morales G, Wiehlmann L, Gudowius P, van Delden C, Tümmler B, Martínez JL, Rojo F 2004 Structure of *Pseudomonas aeruginosa* populations analyzed by single nucleotide polymorphism and pulsed-field gel electrophoresis genotyping. J Bacteriol 186:4228–4237. doi:10.1128/JB.186.13.4228-4237.2004. 15205425 PMC421620

[8] Cho JC, Tiedje JM 2000 Biogeography and degree of endemicity of fluorescent *Pseudomonas* strains in soil. Appl Environ Microbiol 66:5448–5456. doi:10.1128/AEM.66.12.5448-5456.2000. 11097926 PMC92480

[9] Schwartz T, Armant O, Bretschneider N, Hahn A, Kirchen S, Seifert M, Dötsch A 2015 Whole genome and transcriptome analyses of environmental antibiotic sensitive and multi-resistant *Pseudomonas aeruginosa* isolates exposed to waste water and tap water. Microb Biotechnol 8:116–130. doi:10.1111/1751-7915.12156. 25186059 PMC4321378

[10] Brettin T, Davis JJ, Disz T, Edwards RA, Gerdes S, Olsen GJ, Olson R, Overbeek R, Parrello B, Pusch GD, Shukla M, Thomason JA III, Stevens R, Vonstein V, Wattam AR, Xia F 2015 RASTtk: a modular and extensible implementation of the RAST algorithm for building custom annotation pipelines and annotating batches of genomes. Sci Rep 5:8365. doi:10.1038/srep08365. 25666585 PMC4322359

[11] Chuanchuen R, Karkhoff-Schweizer RR, Schweizer HP 2003 High-level triclosan resistance in *Pseudomonas aeruginosa* is solely a result of efflux. Am J Infect Control 31:124–127. doi:10.1067/mic.2003.11. 12665747

[12] Chuanchuen R, Narasaki CT, Schweizer HP 2002 The MexJK efflux pump of *Pseudomonas aeruginosa* requires OprM for antibiotic efflux but not for efflux of triclosan. J Bacteriol 184:5036–5044. doi:10.1128/JB.184.18.5036-5044.2002. 12193619 PMC135324

[13] Mima T, Joshi S, Gomez-Escalada M, Schweizer HP 2007 Identification and characterization of TriABC-OpmH, a triclosan efflux pump of *Pseudomonas aeruginosa* requiring two membrane fusion proteins. J Bacteriol 189:7600–7609. doi:10.1128/JB.00850-07. 17720796 PMC2168734

[14] Lin J, Michel LO, Zhang Q 2002 CmeABC functions as a multidrug efflux system in *Campylobacter jejuni*. Antimicrob Agents Chemother 46:2124–2131. doi:10.1128/AAC.46.7.2124-2131.2002. 12069964 PMC127319

[15] Silver S, Phung LT 1996 Bacterial heavy metal resistance: new surprises. Annu Rev Microbiol 50:753–789. doi:10.1146/annurev.micro.50.1.753. 8905098

[16] Zankari E, Hasman H, Cosentino S, Vestergaard M, Rasmussen S, Lund O, Aarestrup FM, Larsen MV 2012 Identification of acquired antimicrobial resistance genes. J Antimicrob Chemother 67:2640–2644. doi:10.1093/jac/dks261. 22782487 PMC3468078

